# Recombinase polymerase amplification assay for rapid detection of lumpy skin disease virus

**DOI:** 10.1186/s12917-016-0875-5

**Published:** 2016-11-02

**Authors:** Mohamed A. Shalaby, Ayman El-Deeb, Mohamed El-Tholoth, Donata Hoffmann, Claus-Peter Czerny, Frank T. Hufert, Manfred Weidmann, Ahmed Abd El Wahed

**Affiliations:** 1 Virology Department, Faculty of Veterinary Medicine, Cairo University, 12211 Giza, Egypt; 2 Virology Department, Faculty of Veterinary Medicine, Mansoura University, 35516 Mansoura, Egypt; 3 Institute of Diagnostic Virology, Friedrich-Loeffler-Institute, 17493 Greifswald-Insel Riems, Germany; 4 Division of Microbiology and Animal Hygiene, Department of Animal Sciences, Faculty of Agriculture Sciences, Georg-August-University, 37077 Goettingen, Germany; 5 Institute of Microbiology & Virology, Brandenburg Medical School Fontane, 01968 Senftenberg, Germany; 6 Institute of Aquaculture, University of Stirling, FK9 4LA Stirling, Scotland UK

**Keywords:** Lumpy skin disease virus, Recombinase polymerase amplification assay, Point of need test, Cattle

## Abstract

**Background:**

Lumpy skin disease virus (LSDV) is a *Capripoxvirus* infecting cattle and Buffalos. Lumpy skin disease (LSD) leads to significant economic losses due to hide damage, reduction of milk production, mastitis, infertility and mortalities (10 %). Early detection of the virus is crucial to start appropriate outbreak control measures. Veterinarians rely on the presence of the characteristic clinical signs of LSD. Laboratory diagnostics including virus isolation, sequencing and real-time polymerase chain reaction (PCR) are performed at well-equipped laboratories. In this study, a portable, simple, and rapid recombinase polymerase amplification (RPA) assay for the detection of LSDV-genome for the use on farms was developed.

**Results:**

The LSDV RPA assay was performed at 42 °C and detected down to 179 DNA copies/reaction in a maximum of 15 min. Unspecific amplification was observed with neither LSDV-negative samples (*n* = 12) nor nucleic acid preparations from orf virus, bovine papular stomatitis virus, cowpoxvirus, Peste des petits ruminants and Blue tongue virus (serotypes 1, 6 and 8). The clinical sensitivity of the LSDV RPA assay matched 100 % (*n* = 22) to real-time PCR results. In addition, the LSDV RPA assay detected sheep and goat poxviruses.

**Conclusion:**

The LSDV RPA assay is a rapid and sensitive test that could be implemented in field or at quarantine stations for the identification of LSDV infected case.

**Electronic supplementary material:**

The online version of this article (doi:10.1186/s12917-016-0875-5) contains supplementary material, which is available to authorized users.

## Background

Lumpy skin disease (LSD) affects primarily cattle and occasionally buffalo [1, 2]. It causes pyrexia, generalized skin and pox lesions of internal organs, as well as generalized lymphadenopathy [3, 4]. The disease exists in three forms, acute, subacute or unapparent [5]. LSD is caused by an enveloped double-stranded DNA virus called LSD virus (LSDV), which together with sheep poxvirus (SPV) and goat poxvirus (GPV) constitutes the genus *Capripoxvirus* of the *Chordopoxvirinae* subfamily of the *Poxviridae* family [6, 7].

The origin of LSDV is unknown. It was reported for the first time in Zambia in 1929 as a hypersensitivity reaction of cattle to insect bites [8, 9]. In Egypt, LSDV was first reported in Suez and Ismailia Governorates in May and October 1988 and thereafter spread throughout Egypt leading to 50,000 infected cattle and 1,449 mortalities in 1998 [10, 11]. During epizootics LSDV is mainly transmitted mechanically by blood feeding insects e.g. *Aedes aegypti* [12]. Due to the rapid spread of LSDV and the severe economic losses caused, the Office International des Epizooties (OIE) includes LSDV in the listed notifiable disease of cattle [13].

Diagnosis of LSD depends initially on clinical signs. Definite diagnosis can be performed *via* virus isolation, electron microscopy, identification of antigen by immunofluorescence, serum neutralization, agar gel precipitation, antigen capture ELISA and Dot ELISA [3, 14]. In addition, conventional and real-time polymerase chain reactions (PCR) for the detection of the LSDV have been described [3, 15–18]. All the above-mentioned methods are not suitable for screening cattle under field conditions or at quarantine stations, as they require highly skilled staff and a well-equipped laboratory. Simple, portable, and rapid tests to detect LSDV at the point of need could improve initiation of control measures as early as possible. This study describes the development and evaluation of a real-time RPA assay for the detection of LSDV genome.

## Methods

### DNA molecular standards

To produce a molecular LSDV DNA standard, a 910 nt fragment of the G-protein-coupled chemokine receptor (GPCR) gene (6981-7891 of the Genbank accession number: AF325528.1) of the LSDV reference strain (Neethling strain provided by the Pirbright Institute to the Friedrich-Loeffler-Institute, Greifswald-Insel Riems, Germany) was amplified using the in-house designed forward primer (FP): 5′-CATAGTCGATATCCCACATTG-3′, the reverse primer (RP): 5′- GCTAATACTACCAGCACTAC-3′ and the Taq DNA Polymerase (5 PRIME GmbH, Hilden, Germany). The PCR temperature profile was as follows: initial activation at 95 °C/3 min, 30 cycles of 94 °C/60 s, 55 °C/60 s and 72 °C/60 s and a final extension step of 72 °C/5 min. The amplified fragment was ligated into pCR®II using the TA-cloning kit dual promoter and transformed into One shot® chemically competent *E.coli* (Invitrogen, Darmstadt, Germany). Purified plasmids were verified by sequencing (Seqlab, Goettingen, Germany). The plasmid was linearized using the FastDigest HindIII (Fisher Scientific GmbH, Schwerte, Germany). The number of DNA molecules per microliter was measured by the Quant-iT™ PicoGreen® dsDNA Assay Kit (Fisher Scientific GmbH, Schwerte, Germany). Then the DNA standard was diluted to achieve a concentration range of 10^7^–10^1^ DNA molecules/μl. The standard was tested by applying primers and a modified probe (FP, 5′-GATAGTATCGCTAAACAATGG-3; RP, 5′-ATCCAAACCACCATACTAAG-3′; P, 5′-FAM-ACCTAGCTGTAGTTCACCCAGTAAA-TAMRA-3′) of a published real-time PCR protocol [16] using the Light Cycler 2.0 and the FastStart DNA Master HybProbe kit (Roche, Manheim, Germany).

### LSDV RPA oligonucleotides and conditions

**Fig. 1:**
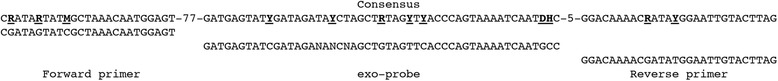
Alignment of the LSDV RPA primers and exo-probe sequences with the consensus sequence of 132 *capripoxviruses* GPCR genes downloaded from Genbank (Geneious® 6.1.5, Biomatters Limited, New Zealand). Mismatches are indicated in bold and underlined. NNN are sites of the quencher and fluropohore in following order (BHQ1-dT) (Tetrahydrofuran) (FAM-dT). R is A or G; Y, C or T; M, A or C; D, A or G or T; H, A or C or T

RPA primers and exo probe (Fig. 1) were synthesized by TIB MOLBIOL (Berlin, Germany). The LSDV RPA was performed in a 50 μl volume using the TwistAmp™ exo lyophilized kit (TwistDx, Cambridge, UK), using 420 nM RPA primers and 120 nM RPA exo-probe, 29.5 μl of rehydration buffer, 12.2 of molecular biology grade water. A mastermix was added directly to the lyophilized pellet provided in the tubes of a 8-tubes strip. Thereafter, 2.5 μl of Mg acetate (1 mM) were added to each lid. Finally, one microliter of DNA template was added to the pellet. The tube was closed, centrifuged, mixed well and centrifuged again before placed into the tubescanner (Twista, TwistDx, Cambridge, UK) for 15 min at 42 °C. After 230 s, the strip was retrieved, vortexed, centrifuged and placed again into the tubescanner (Twista, TwistDx, Cambridge, UK). The fluorescence signal was measured each 20 s using the FAM channel. In each run positive and negative controls were included. A combined threshold and 1^st^ derivative analysis was used for signal interpretation. Samples produced an exponential amplification curve above the threshold of the negative control were consider positive.

### LSDV assay cross reactivity

The Friedrich-Loeffler-Institute, Greifswald-Insel Riems, Germany provided reference nucleic acids for LSDV, SPV, GPV, orf virus, bovine papular stomatitis virus, cowpoxvirus, Peste des petits ruminants and Blue tongue virus (serotypes 1, 6 and 8) (Table 1). All samples contained a high concentration of viral nucleic acid as determined by the respective real-time PCRs recommended by the OIE (CT: 12-20).

**Table 1:** List of reference viral strains

Virus	Strain	Reference
Lumpy skin disease virus	Neethling strain	[40]
Sheep poxvirus	Russia	NA
Goat poxvirus	Indian	NA
Cowpoxvirus	2	[41]
Orf Virus	Burgheßler	[42]
Bovine papular stomatitis virus	M1	[43]
Peste de petite Ruminant Virus	lineage IV_Kurdistan2011	[44]
Blue tongue virus	Serotypes 1, 6 and 8	[45]

*NA* is non-applicable

### Clinical sensitivity and specificity of the LSDV RPA assay

Twenty-two skin nodules of suspected LSDV-infected cattle were collected during the summer of 2012 in Dakahlia Governorate, Egypt. Diseased cattle exhibited either localized or generalized multiple skin nodules with or without systemic signs.

DNA was extracted from the twenty-two skin nodules and twelve skin samples from apparently healthy cows using QIAamp DNA Mini Kit (Qiagen, Hilden, Germany). All samples were screened simultaneously by the LSDV RPA assay and real-time PCR as described above. The real time PCR CT values for these samples ranged from CT 18 to 35 (Additional file 1: Table S1).

### Statistical methods

For the determination of the LSDV RPA assay analytical sensitivity by the molecular DNA standard, a semi-log regression analysis (PRISM, Graphpad Software Inc., San Diego, California) and a probit analysis (STATISTICA, StatSoft, Hamburg, Germany) were performed by plotting the RPA threshold time against the number of molecules detected. Clinical sensitivity and specificity values were calculated using standard formulas.

## Results

**Fig. 2:**
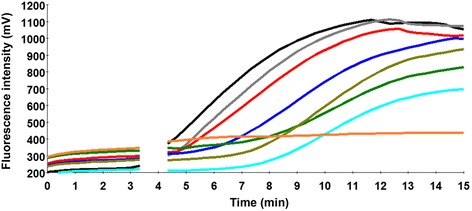
The results layout of one LSDV RPA assay run. Fluorescence development over time using a dilution range of 10^7^–10^1^ molecules/μl of the DNA molecular standard (Graph generated by ESEquant tubescanner software). 10^7^ represented by black line; 10^6^, gray; 10^5^, red; 10^4^, blue; 10^3^, green; 10^2^, cyan; 10^1^, dark khaki; negative control, orange. The LSDV RPA assay detected down to 10 DNA molecules/reaction. After 230 s, the strip was taken out of the tubescanner for mixing, therefore, no fluorescence signals were recorded

**Fig. 3:**
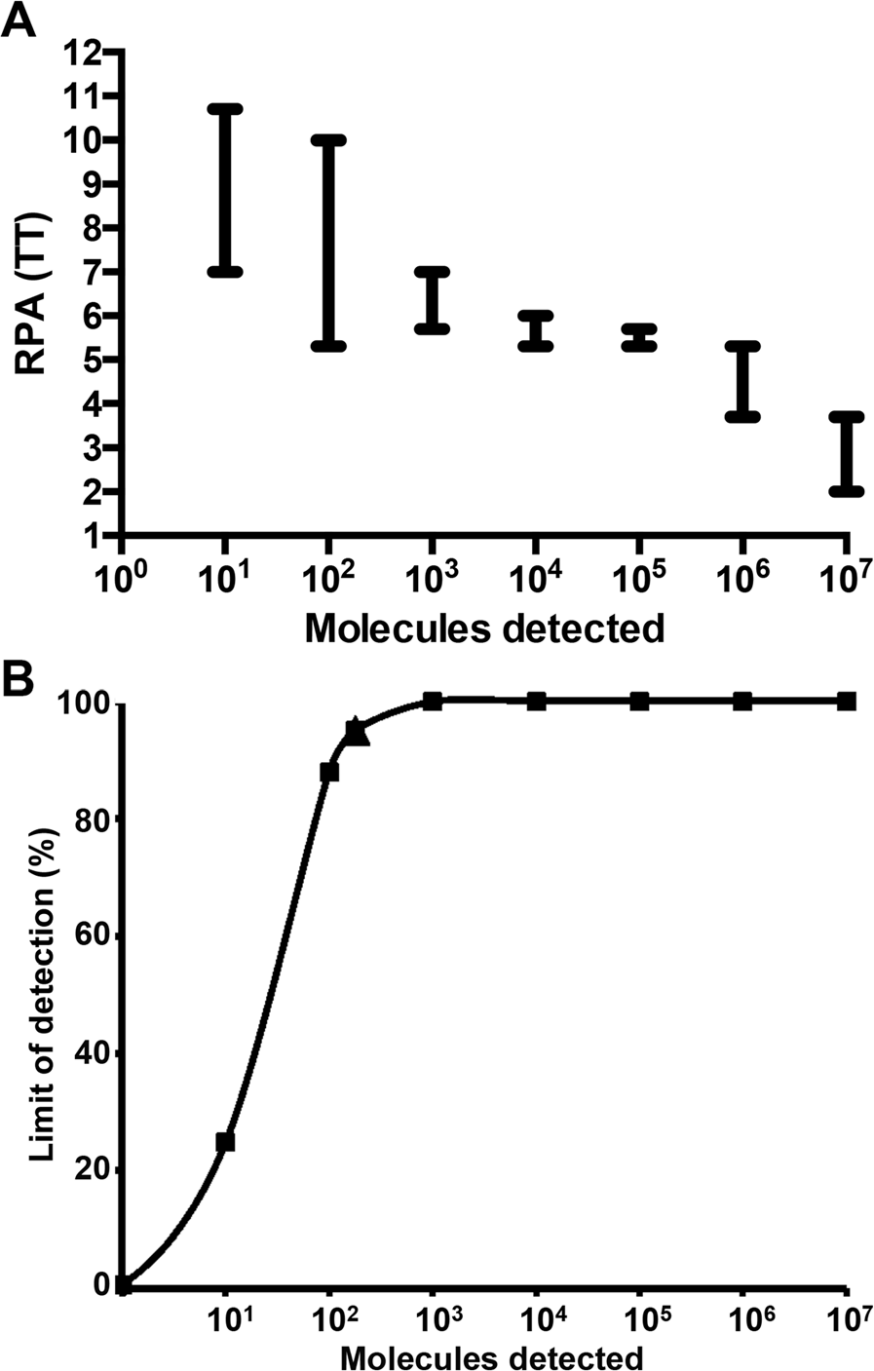
Performance of the LSDV RPA assay using data set of eight RPA assay runs. **a** Semi-logarithmic regression (**b**) Probit regression analysis. The LSDV RPA assay yielded results between 2–12 min. The results of the 10^7^–10^3^ were consistence, therefore no error bars was included (**a**). The limit of detection (179 DNA molecules) at 95 % probability was is depicted by a triangle (**b**)

The LSDV GPCR gene plasmid standard was used to determine the analytical sensitivity of the assay using a dilution range between 10^7^–10^1^/μl (Fig. 2). The LSDV RPA assay was performed eight times on the molecular standard, in which 10^7^–10^3^ DNA molecules were detected in 8/8 runs, 10^2^, 7/8 and 10^1^, 2/8 (Fig. 3a, Additional file 2: Table S2). Due to the inconsistency in the results, a probit regression analysis was applied, in which the sensitivity in 95 % of cases was determined at 179 DNA molecules/reaction (Fig. 3b). While the real-time PCR analytical sensitivity was 37 DNA copies/reaction [16].

The LSDV RPA assay showed no cross detection with orf virus, bovine papular stomatitis virus, cowpoxvirus, Peste des petits ruminants and Blue tongue virus (serotypes 1, 6 and 8) genome. SPV and GPV, however, were detected by the LSDV RPA assay as well. The LSDV RPA assay was validated using 12 negative skin samples and 22 LSDV positive skin nodule samples. In comparison to real-time PCR, clinical sensitivity and specificity of the LSDV RPA assay was 100 % (Additional file 1: Table S1).

## Discussion

Veterinarians rely mainly on the appearance of clinical signs for LSD diagnosis [8]. Establishment of a rapid diagnostic test to identify early stages of an LSD outbreak would allow rapid execution of control measures.

The developed LSDV RPA assay was highly sensitive (179 DNA copies detected/reaction) and rapid (total time: 2–15 min). In addition, we were able to obtain the same clinical sensitivity and specificity as the well-established real-time PCR assay [16] when testing 22 skin samples. The RPA assay is a technique for the isothermal amplification of DNA using enzymes and proteins to replace the repetitive cycles of three temperatures used for PCR [19]. Thus, RPA can be operated by portable more simple heating and detection devices instead of using thermal cycler devices. Moreover, RPA reagents are cold chain independent [20–22], which make them ideal for point of need testing.

The LSDV RPA exo-probe was designed to detect LSDV by placing two mismatches to the sequences of SPV and GPV at its 3′ prime end (Fig. 1 and Additional file 3: Figure S1) as well as the primers amplified 186 of the most variable gene in the *capripoxviruses*, GPCR gene [23, 24]. Nevertheless, SPV and GPV were detected. This might be due to the fact that the length (48–52 bp) of the RPA exo probe compensates for the presence of nucleotide mismatches [25, 26]. Also, the position of mismatches appear not to affect RPA oligonucleotide binding [27], but have a great influence on the PCR primes and probe [28]. However, the assay will be also useful for detecting SPV and GPV, unfortunately, no clinical samples were available to validate the assay for both viruses.

Several conventional and real-time PCRs have been established to identify the *capripoxviruses* [18, 29–34], but none are able to distinguish between various species [35]. One PCR assay was established for the differentiation between these viruses; however, many GPVs were identified as SPV [16]. This is due to the high homology of up to 96 % between the members of this genus [23], which also affected the LSDV RPA assay. The OIE recommends sequencing and phylogenetic analysis to differentiate between LSDV, SPV and GPV. For a point of need field test the LSDV-RPA described here can still be of help as SPV and GPV are not known to infect cattle and using it on cattle samples, therefore, provides the specificity needed especially as the panel of other cattle infecting viruses tested all scored negative in the cross detection assessment.

Several loop-mediated isothermal amplification (LAMP) assays have been established to identify *capripoxviruses* [36–38]. The design of LAMP assays requires four to six oligonucleotides and a minimum of four binding sites. The LAMP results can be read by naked eye, if turbidity read out is used [39] after about 60 min. In contrast, the RPA assay developed here was very fast (15 min) and required two primers and one probe.

## Conclusion

In conclusion, LSDV RPA yielded similar results as the corresponding real-time PCR assay, but RPA was quicker and much easier to handle. Furthermore, combination with a simple extraction method will allow its employment at low resource settings, quarantine stations or farms.

## Additional files

Additional file 1: Table S1. Screening 22 skin nodules samples with real-time PCR and RPA assays. (DOCX 57 kb)
Additional file 2: Table S2. Reproducibility of LSDV RPA assay using data sets of eight RPA assay runs using the DNA molecular standards. 10^7^–10^3^ DNA molecules were detected 8 out of 8 runs; 10^2^, 7/8 and 10^1^, 2/8. (DOCX 54 kb)
Additional file 3: Figure S1. Mapping 132 nucleotide sequences derived by BLAST nucleotide search to the LSDV RPA amplicon as well as RPA primers and probe. The alignment was performed by using Geneious (V: 9.0.5. Biomatters Limited. New Zealand). The Genbank accession number and name were given. Grey represents the identical sequence. A. C. G. T were highlighted in red. violet. yellow. green. respectively. whenever a mismatch to the LSDV RPA amplicon was recorded. (DOCX 515 kb)

## Abbreviations

FP: Forward primer
GPCR: G-protein-coupled chemokine receptor
GPV: Goat poxvirus
LAMP: Loop-mediated isothermal amplification
LSD: Lumpy skin disease
LSDV: Lumpy skin disease virus
n: Number
OIE: Office International des Epizooties
PCR: Polymerase chain reaction
RP: Reverse primer
RPA: Recombinase polymerase amplification
SPV: Sheep poxvirus

## References

[1] Sharawi SS, Abd El-Rahim IH (2011). The utility of polymerase chain reaction for diagnosis of lumpy skin disease in cattle and water buffaloes in Egypt. Rev Sci Tech.

[2] El-Tholoth M, El-Kenawy AA. G-Protein-Coupled Chemokine Receptor Gene in Lumpy Skin Disease Virus Isolates from Cattle and Water Buffalo (Bubalus bubalis) in Egypt. Transbound Emerg Dis. 2016;63(6):e288-e295. 10.1111/tbed.12344 25754131

[3] Tuppurainen ES, Venter EH, Coetzer JA (2005). The detection of lumpy skin disease virus in samples of experimentally infected cattle using different diagnostic techniques. Onderstepoort J Vet Res.

[4] Davies FG (1991). Lumpy skin disease of cattle: A growing problem in Africa and the Near East. World Animal Review.

[5] Weiss KE (1968). Lumpy skin disease. Monogr Virol.

[6] Buller RM, Arif BM, Black DN, Dumbell KR, Esposito JJ, Lefkowitz EJ, McFadden G, Moss B, Mercer AA, Moyer RW, Fauquet CM, Mayo MA, Maniloff J, Desselberger U, Ball LA (2005). Family Poxviridae. Virus Taxonomy: Classification and Nomenclature of Viruses.

[7] Diallo A, Viljoen GJ (2007). Genus Capripoxvirus.

[8] Weiss KE (1968). Lumpy Skin Disease Virus. Cytomegaloviruses Rinderpest Virus Lumpy Skin Disease Virus.

[9] Morris JPA. Pseudo-urticaria. Northern Rhodesia. Dept Anim Health Ann Rpt. 1930:12.

[10] Salib FA, Osman AH (2011). Incidence of lumpy skin disease among Egyptian cattle in Giza Governorate, Egypt. Veterinary World.

[11] Ali AA, Esmat M, Attia H, Selim A, Abdel-Hamid YM (1990). Clinical and pathological studies on lumpy skin disease in Egypt. Vet Rec.

[12] Chihota CM, Rennie LF, Kitching RP, Mellor PS (2001). Mechanical transmission of lumpy skin disease virus by Aedes aegypti (Diptera: Culicidae). Epidemiology & Infection.

[13] Bowden TR, Babiuk SL, Parkyn GR, Copps JS, Boyle DB (2008). Capripoxvirus tissue tropism and shedding: A quantitative study in experimentally infected sheep and goats. Virology.

[14] OIE (2010). Lumpy Skin Disease. OIE Terrestrial Manual, vol. Chapter 2.4.14.

[15] Armson B, Fowler VL, Tuppurainen ES, Howson EL, Madi M, Sallu R, Kasanga CJ, Pearson C, Wood J, Martin P, et al. Detection of Capripoxvirus DNA Using a Field-Ready Nucleic Acid Extraction and Real-Time PCR Platform. Transbound Emerg Dis. 2015. 10.1111/tbed.12447 PMC5434827 26608662

[16] Lamien CE, Lelenta M, Goger W, Silber R, Tuppurainen E, Matijevic M, Luckins AG, Diallo A (2011). Real time PCR method for simultaneous detection, quantitation and differentiation of capripoxviruses. J Virol Methods.

[17] Menasherow S, Rubinstein-Giuni M, Kovtunenko A, Eyngor Y, Fridgut O, Rotenberg D, Khinich Y, Stram Y (2014). Development of an assay to differentiate between virulent and vaccine strains of lumpy skin disease virus (LSDV). J Virol Methods.

[18] Balinsky CA, Delhon G, Smoliga G, Prarat M, French RA, Geary SJ, Rock DL, Rodriguez LL (2008). Rapid preclinical detection of sheeppox virus by a real-time PCR assay. J Clin Microbiol.

[19] Piepenburg O, Williams CH, Stemple DL, Armes NA. DNA detection using recombination proteins. PLoS Biol. 2006;4, e204. http://journals.plos.org/plosbiology/article?id=10.1371/journal.pbio.0040204. 10.1371/journal.pbio.0040204 PMC1475771 16756388

[20] Abd El Wahed A, Patel P, Heidenreich D, Hufert FT, Weidmann M. Reverse transcription recombinase polymerase amplification assay for the detection of middle East respiratory syndrome coronavirus. PLoS Curr. 2013;5. http://currents.plos.org/outbreaks/article/reverse-transcription-recombinase-polymeraseamplification-assay-for-the-detection-of-middle-east-respiratory-syndrome-coronavirus/. 10.1371/currents.outbreaks.62df1c7c75ffc96cd59034531e2e8364 PMC3871419 24459611

[21] Abd El Wahed A, Weidmann M, Hufert FT (2015). Diagnostics-in-a-Suitcase: Development of a portable and rapid assay for the detection of the emerging avian influenza A (H7N9) virus. J Clin Virol.

[22] Faye O, Faye O, Soropogui B, Patel P, El Wahed AA, Loucoubar C, Fall G, Kiory D, Magassouba N, Keita S, et al. Development and deployment of a rapid recombinase polymerase amplification Ebola virus detection assay in Guinea in 2015. Euro Surveill. 2015;20. http://www.eurosurveillance.org/ViewArticle.aspx?ArticleId=21289. 10.2807/1560-7917.ES.2015.20.44.30053 26558690

[23] Tulman ER, Afonso CL, Lu Z, Zsak L, Sur JH, Sandybaev NT, Kerembekova UZ, Zaitsev VL, Kutish GF, Rock DL (2002). The genomes of sheeppox and goatpox viruses. J Virol.

[24] Tulman ER, Afonso CL, Lu Z, Zsak L, Kutish GF, Rock DL (2001). Genome of lumpy skin disease virus. J Virol.

[25] Abd El Wahed A, El-Deeb A, El-Tholoth M, Abd El Kader H, Ahmed A, Hassan S, Hoffmann B, Haas B, Shalaby MA, Hufert FT, Weidmann M (2013). A portable reverse transcription recombinase polymerase amplification assay for rapid detection of foot-and-mouth disease virus. PLoS One.

[26] Boyle DS, Lehman DA, Lillis L, Peterson D, Singhal M, Armes N, Parker M, Piepenburg O, Overbaugh J. Rapid Detection of HIV-1 Proviral DNA for Early Infant Diagnosis Using Recombinase Polymerase Amplification. MBio. 2013;4. 10.1128/mBio.00135-13 PMC3622927 23549916

[27] Amer HM, Abd El Wahed A, Shalaby MA, Almajhdi FN, Hufert FT, Weidmann M (2013). A new approach for diagnosis of bovine coronavirus using a reverse transcription recombinase polymerase amplification assay. J Virol Methods.

[28] Whiley DM, Sloots TP (2006). Sequence variation can affect the performance of minor groove binder TaqMan probes in viral diagnostic assays. J Clin Virol.

[29] Cheng Z, Yue J, Li Y, Xu L, Wang K, Zhou B, Chen J, Li J, Jiang N (2009). Development and application of TaqMan-MGB real-time quantitative PCR assay for detection of goat pox virus. Sheng Wu Gong Cheng Xue Bao.

[30] Haegeman A, Zro K, Vandenbussche F, Demeestere L, Van Campe W, Ennaji MM, De Clercq K (2013). Development and validation of three Capripoxvirus real-time PCRs for parallel testing. J Virol Methods.

[31] Tian H, Wu J, Chen Y, Zhang K, Shang Y, Liu X (2012). Development of a SYBR green real-time PCR method for rapid detection of sheep pox virus. Virol J.

[32] Venkatesan G, Balamurugan V, Bhanuprakash V (2014). TaqMan based real-time duplex PCR for simultaneous detection and quantitation of capripox and orf virus genomes in clinical samples. J Virol Methods.

[33] Zro K, Azelmat S, Bendouro Y, Kuhn JH, El Fahime E, Ennaji MM (2014). PCR-based assay to detect sheeppox virus in ocular, nasal, and rectal swabs from infected Moroccan sheep. J Virol Methods.

[34] Markoulatos P, Mangana-Vougiouka O, Koptopoulos G, Nomikou K, Papadopoulos O (2000). Detection of sheep poxvirus in skin biopsy samples by a multiplex polymerase chain reaction. J Virol Methods.

[35] OIE (2010). Sheep pox and goat pox. OIE Terrestrial Manual 2010.

[36] Das A, Babiuk S, McIntosh MT (2012). Development of a loop-mediated isothermal amplification assay for rapid detection of capripoxviruses. J Clin Microbiol.

[37] Venkatesan G, Balamurugan V, Bhanuprakash V, Singh RK, Pandey AB. Loop-mediated isothermal amplification assay for rapid and sensitive detection of sheep pox and goat pox viruses in clinical samples. Mol Cell Probes. 2016;30(3):174-7. 10.1016/j.mcp.2016.02.004 26872529

[38] Zhao Z, Fan B, Wu G, Yan X, Li Y, Zhou X, Yue H, Dai X, Zhu H, Tian B (2014). Development of loop-mediated isothermal amplification assay for specific and rapid detection of differential goat pox virus and sheep pox virus. BMC Microbiol.

[39] Notomi T, Okayama H, Masubuchi H, Yonekawa T, Watanabe K, Amino N, Hase T (2000). Loop-mediated isothermal amplification of DNA. Nucleic Acids Res.

[40] Gari G, Abie G, Gizaw D, Wubete A, Kidane M, Asgedom H, Bayissa B, Ayelet G, Oura CA, Roger F, Tuppurainen ES (2015). Evaluation of the safety, immunogenicity and efficacy of three capripoxvirus vaccine strains against lumpy skin disease virus. Vaccine.

[41] Liebermann H, Schulze P, Liebermann H (1967). Euterpocken in Deutschland. Archiv für Experimentelle Veterinärmedi.

[42] Schmidt D (1967). Experimentelle Beiträge zur Kenntnis der Dermatitis pustulosa des Schafes.1. Versuche zur Reinigung des Virus der Dermatitis pustulosa und zur Abtrennung des komplementbindenden Antigens von den Elementarkörpern. Archiv für Experimentelle Veterinärmedi.

[43] Liebermann H, Urbaneck D (1966). Isolierung und Identifizierung von Stomatitis-papulosa Virus mit Hilfe der Zellkultur. Archiv für Experimentelle Veterinärmedi.

[44] Hoffmann B, Wiesner H, Maltzan J, Mustefa R, Eschbaumer M, Arif FA, Beer M (2012). Fatalities in wild goats in Kurdistan associated with Peste des Petits Ruminants virus. Transbound Emerg Dis.

[45] Hoffmann B, Eschbaumer M, Beer M (2009). Real-time quantitative reverse transcription-PCR assays specifically detecting bluetongue virus serotypes 1, 6, and 8. J Clin Microbiol.

