# Supplementary material for: Recombinase polymerase amplification assay for rapid detection of lumpy skin disease virus
Source: BMC Vet Res. 2016 Nov 2;12:244. doi: 10.1186/s12917-016-0875-5 (PMC5094145; doi:10.1186/s12917-016-0875-5)
Supplement: Additional file 1: Table S1. — Screening 22 skin nodules samples with real-time PCR and RPA assays. (DOCX 57 kb) [file 12917_2016_875_MOESM1_ESM.docx]

**Table S1. Screening 22 skin nodules samples with real-time PCR and RPA assays.**

| **Sample ID** | **Real-time PCR**  **(CT Value)** | **RPA**  **(TT- min)** |
| --- | --- | --- |
| 1 | 18.98 | 3.7 |
| 2 | 19.44 | 5.3 |
| 3 | 21.44 | 5.3 |
| 4 | 21.96 | 5.3 |
| 5 | 24.5 | 5.3 |
| 6 | 24.89 | 5.3 |
| 7 | 25 | 14 |
| 8 | 26.17 | 5.3 |
| 9 | 26.44 | 5.7 |
| 10 | 26.59 | 5.7 |
| 11 | 28.88 | 5.7 |
| 12 | 29.63 | 6.7 |
| 13 | 29.88 | 6 |
| 14 | 30.83 | 6 |
| 15 | 31.29 | 5.7 |
| 16 | 31.92 | 3 |
| 17 | 32 | 6 |
| 18 | 32.34 | 7 |
| 19 | 33.48 | 5.3 |
| 20 | 33.84 | 5.7 |
| 21 | 34.45 | 5.7 |
| 22 | 35.98 | 7.7 |
