# Supplementary material for: Recombinase polymerase amplification assay for rapid detection of lumpy skin disease virus
Source: BMC Vet Res. 2016 Nov 2;12:244. doi: 10.1186/s12917-016-0875-5 (PMC5094145; doi:10.1186/s12917-016-0875-5)
Supplement: Additional file 2: Table S2. — Reproducibility of LSDV RPA assay using data sets of eight RPA assay runs using the DNA molecular standards. 107–103 DNA molecules were detected 8 out of 8 runs; 102, 7/8 and 101, 2/8. (DOCX 54 kb) [file 12917_2016_875_MOESM2_ESM.docx]

**Table S2. Reproducibility of LSDV RPA assay using data sets of eight RPA assay runs using the DNA molecular standards. 10^7^-10^3^ DNA molecules were detected 8 out of 8 runs; 10^2^, 7/8 and 10^1^, 2/8.**

| **Conc.** | **Run (threshold time in minutes)** | | | | | | | |
| --- | --- | --- | --- | --- | --- | --- | --- | --- |
|  | **1** | **2** | **3** | **4** | **5** | **6** | **7** | **8** |
| 10^1^ | Neg | 10.70 | Neg | Neg | Neg | Neg | Neg | 7.00 |
| 10^2^ | 5.30 | 7.70 | 8.70 | 9.30 | 10.00 | 8.00 | Neg | 7.30 |
| 10^3^ | 5.70 | 7.00 | 7.00 | 6.70 | 6.00 | 5.70 | 6.00 | 5.70 |
| 10^4^ | 5.30 | 5.30 | 5.30 | 5.70 | 5.70 | 5.30 | 6.00 | 5.70 |
| 10^5^ | 5.30 | 5.30 | 5.30 | 5.70 | 5.30 | 5.30 | 5.30 | 5.30 |
| 10^6^ | 3.70 | 5.30 | 5.30 | 5.30 | 5.30 | 5.30 | 5.30 | 5.30 |
| 10^7^ | 2.00 | 3.30 | 3.70 | 3.30 | 3.30 | 3.00 | 3.30 | 3.30 |
