# Supplementary material for: Recombinase polymerase amplification assay for rapid detection of lumpy skin disease virus
Source: BMC Vet Res. 2016 Nov 2;12:244. doi: 10.1186/s12917-016-0875-5 (PMC5094145; doi:10.1186/s12917-016-0875-5)
Supplement: Additional file 3: Figure S1. — Mapping 132 nucleotide sequences derived by BLAST nucleotide search to the LSDV RPA amplicon as well as RPA primers and probe. The alignment was performed by using Geneious (V: 9.0.5. Biomatters Limited. New Zealand). The Genbank accession number and name were given. Grey represents the identical sequence. A. C. G. T were highlighted in red. violet. yellow. green. respectively. whenever a mismatch to the LSDV RPA amplicon was recorded. (DOCX 515 kb) [file 12917_2016_875_MOESM3_ESM.docx]

**Fig S1. Mapping 132 nucleotide sequences derived by BLAST nucleotide search to the LSDV RPA amplicon as well as RPA primers and probe.** The alignment was performed by using Geneious (V: 9.0.5. Biomatters Limited. New Zealand). The Genbank accession number and name were given*.* Grey represents the identical sequence. A. C. G. T were highlighted in red. violet. yellow. green. respectively. whenever a mismatch to the LSDV RPA amplicon was recorded.
